# Case report: Analysis of a gene variant and prenatal diagnosis in a family with megalencephalic leukoencephalopathy with subcortical cysts

**DOI:** 10.3389/fneur.2023.1253398

**Published:** 2023-10-20

**Authors:** Xi Chen, Haibo Qu, Qiang Yao, Xiaotang Cai, Tiantian He, Xuemei Zhang

**Affiliations:** ^1^Department of Medical Genetics and Prenatal Diagnosis Center, West China Second University Hospital, Sichuan University, Chengdu, China; ^2^Department of Obstetrics and Gynecology, West China Second University Hospital, Sichuan University, Chengdu, China; ^3^Key Laboratory of Birth Defects and Related Diseases of Women and Children (Sichuan University), Ministry of Education, Chengdu, China; ^4^Department of Radiology, West China Second University Hospital, Sichuan University, Chengdu, China; ^5^Department of Rehabilitation, West China Second University Hospital, Sichuan University, Chengdu, China

**Keywords:** MLC, *MLC1*, leukodystrophy, splice mutation, clinical phenotype, prenatal diagnosis, genetic counseling

## Abstract

Megalencephalic leukoencephalopathy with subcortical cysts (MLC) is a rare inherited cerebral white matter disorder in children. Pathogenic variations in the causative gene *MLC1* are found in approximately 76% of patients and are inherited in an autosomal recessive manner. In this study, we identified an IVS2 + 1delG variant in *MLC1* in the firstborn girl of a pregnant woman who has the clinical features of MLC, including macrocephaly, motor development delay, progressive functional deterioration, and myelinopathy, whereas no obvious subcortical cysts were observed by magnetic resonance imaging of the brain. The proband is homozygous for the IVS2 + 1delG mutation, which was inherited from the parents. This variant disrupts the donor splice site, causing an abnormal transcript that results in a premature termination codon and produces a truncated protein, which was confirmed to affect splicing by *MLC1* cDNA analysis. This variant was also detected in family members, and a prenatal diagnosis for the fetus was undertaken. Eventually, the couple gave birth to an unaffected baby. Furthermore, we conducted a long-term follow-up of the proband’s clinical course. This report improves our understanding of the genetic and phenotypic characteristics of MLC and provides a new genetic basis for prenatal diagnosis and genetic counseling.

## 1. Introduction

Megalencephalic leukoencephalopathy with subcortical cysts (MLC) is a rare inherited cerebral white matter disorder characterized by early-onset macrocephaly and delayed-onset neurological deterioration, including cerebellar ataxia, spasticity, epilepsy, and mild cognitive decline (1, 2). Magnetic resonance imaging (MRI) of the brain shows evidence of severe white matter involvement and cysts in the tips of the temporal lobes, as well as in the frontoparietal subcortical area (3). The disease is progressive and results in the regression of motor and cognitive functions.

Two genes are associated with three types of MLC. Type 1 (OMIM 604004) is caused by a recessive mutation in *MLC1* (OMIM 605908), type 2A (OMIM 613925) is caused by a recessive mutation in *HEPACAM* (OMIM 611642), and type 2B (OMIM 613926) is caused by a dominant mutation in *HEPACAM*. Biallelic pathogenic variants in *MLC1* are observed in approximately 76% of individuals with MLC, whereas pathogenic variants in *HEPACAM* are found in approximately 22% (1). This suggests there may be other related genes not yet discovered.

The *MLC1* gene has been mapped to chromosome 22q13.33 (4, 5), which contains 12 exons, encodes 377 amino acids, and contains 8 transmembrane domains (6, 7). Over 100 mutations in *MLC1* have been reported globally (HGMD® Professional 2023.4), most of which occur throughout the coding and non-coding regions. All types of mutations are included: missense and nonsense mutations (51%), splice mutations (19%), deletions (14%), and insertions (6%) (HGMD).

In this study, we recruited a woman in early pregnancy whose firstborn girl was clinically diagnosed with leukoencephalopathy based on symptoms and brain MRI findings. In this family, a splicing mutation was identified in *MLC1*, which induces protein truncation with a premature stop codon. A prenatal diagnosis was made for this family, and clinical follow-up was performed for the proband and second child.

## 2. Materials and methods

### 2.1. Clinical materials and participants

Patients and family members were recruited at the West China Second University Hospital, and blood samples were obtained from all participants. Genomic DNA was extracted from peripheral blood according to the manufacturer’s instructions (CWBIO, Beijing, China), and total RNA was isolated using the RNA Pure Blood Kit (CWBIO). Clinical data were collected and evaluated by a multidisciplinary team of geneticists, radiologists, obstetricians, and pediatricians. The phenotypes of the individuals were longitudinally and systematically evaluated. Studies involving human participants were reviewed and approved by the Medical Ethics Committee of the West China Second University Hospital, Sichuan University. Written informed consent to participate in this study was obtained from participants or their legal guardians.

### 2.2. Mutation identification and analysis

Exome sequencing of the target gene panel associated with leukoencephalopathy was performed on the proband’s DNA sample at Beijing Mygenostics Inc. (Beijing, P.R. China) using an Illumina NextSeq 500 (Illumina, San Diego, CA, USA). The mutation was confirmed by Sanger sequencing. All family members and 100 Chinese control samples were screened for this mutation. The primers for Sanger sequencing can be provided upon request.

The mRNA transcription of *MLC1* was examined using reverse transcription-polymerase chain reaction (RT-PCR) (PrimeScript™ RT reagent Kit, TaKaRa). The following were the primers used: forward (5′-AAC TGG TGA CAC GTG GCT GT-3′), reverse (5′-TTG CTG ATG GGT TCA GGA CT-3′), or reverse (5′-ACT TCG TCC AGA ATG TTG GC-3′). Both PCR and RT-PCR were performed in a 25-μL reaction, containing a 50 ng template, 12.5 μL Gold Taq Green Master Mix (Promega, Madison, Wisconsin, USA), and 10 pmol/L of each primer. The reactions were performed under the following conditions: 95°C for 1.5 min, 35 cycles at 94°C for 40 s, 60°C for 40 s, and 72°C for 40 s, followed by a final extension at 72°C for 5 min. The amplifiers were purified using the Wizard SV Gel and PCR Clean-Up System (Promega), followed by direct sequencing on an ABI 3500 Genetic Analyzer (Applied Biosystems, Waltham, MA, USA), and the data was evaluated using Chromas software (2.6.5). Alphafold 2^1^ was used to predict and analyze protein structure.

### 2.3. Prenatal diagnosis

Amniocentesis was performed at 19 weeks of gestation, according to standard clinical operating procedures (8). Amniotic fluid was extracted from the gravida for prenatal diagnosis to identify whether the second fetus shared the mutation found in the proband. After high-speed centrifugation (900 rcf, 8 min) of the amniotic fluid, the supernatant was discarded and the bottom cell precipitate was retained. DNA was extracted and sequenced, as described above. The mother’s blood sample was collected in an EDTA vacutainer to identify maternal cell contamination during prenatal diagnosis.

## 3. Results

### 3.1. Case presentation

**Figure 1:**
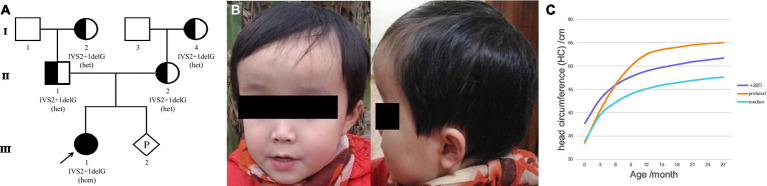
Family pedigree and clinical features of the proband. **(A)** Family pedigree. The proband was noted to have a homozygous variant. Parents and grandparents carried the heterozygous variant. The fetus carried the wild-type form of *MLC1*. **(B)** The appearance of the proband’s head at 2 years of age. **(C)** The curve of the HC of the proband. Macrocephaly was observed during her first year of life and her HC at 15 months was 53.5 cm, > +3SD. The early rapid growth of the head, crossing +3 SD at approximately 6 months, was followed only a few months later (at approximately 1 year of age) by a slow growth that parallels the median curve.

A 29-year-old pregnant woman, gravida 2, para 1, was referred to the Department of Medical Genetics and Prenatal Diagnosis Center of West China Second University Hospital because her firstborn was clinically suspected of having leukoencephalopathy. She and her spouse were non-consanguineous, with no notable medical conditions, and denied a family history of genetic diseases or a known history of neurological disorders (Figure 1A). She was in her second pregnancy at 12 weeks with no complications and had no notable history in her first pregnancy.

**Figure 2:**
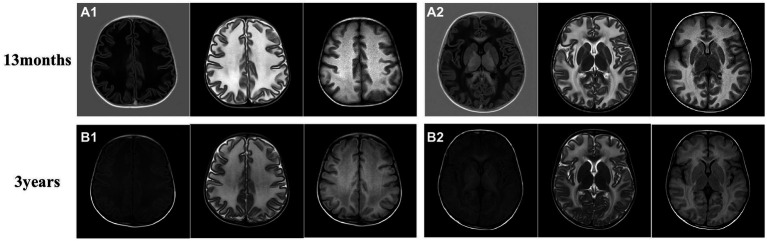
Brain magnetic resonance imaging (MRI) of the proband. **(A)** The brain MRI of the proband at 13 months. **(B)** Another image was taken at 3 years old. The MRI revealed swelling in the bilateral cerebral cortex and diffuse signal abnormalities in cerebral white matter, showing a low signal on T1-weighted images and a high signal on T2-weighted images, as well as in fluid-attenuated inversion recovery images. Subcortical cysts were not observed in any section.

The proband (firstborn), a 2-year-old girl, was born naturally at full term and had a normal occipitofrontal circumference (OFC) (33.5 cm) and weight (3.2 kg) at birth. Macrocephaly was observed during her first year of life when she appeared with a large head circumference (HC) (46 cm, > +3 SD) at 6 months of age [according to the median HC (MHC) in Chinese girls (9)] and presented an HC of 48.5 cm (> +3 SD, MHC: 43.5 cm) at 8 months of age. At the age of 1 year and 3 months, her HC increased to 53.5 cm (> +3 SD, MHC: 45.9 cm; Figures 1B,C), whereas her height (75 cm) and weight (10 kg) were normal. Brain MRI at 13 months revealed swelling in the bilateral cerebral cortex and diffuse signal abnormalities in the cerebral white matter, showing a low signal on T1-weighted images and a high signal on T2-weighted images, as well as fluid-attenuated inversion recovery images; however, subcortical cysts were not seen in all sections (Figure 2A).

**Figure 3:**
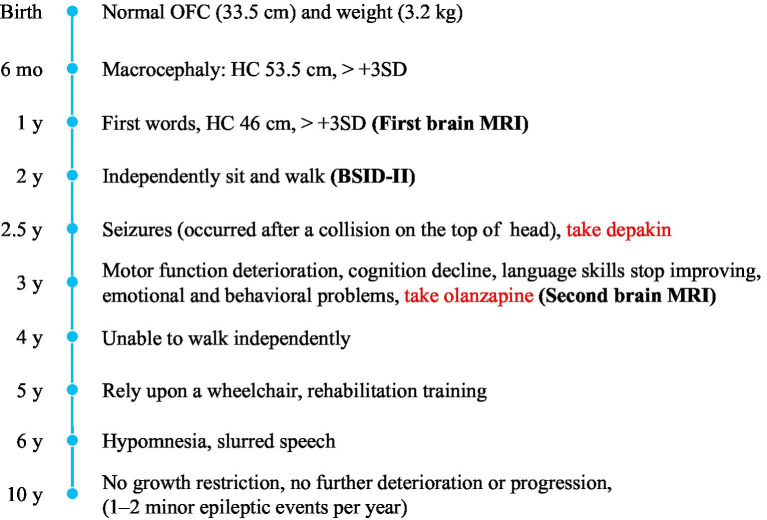
Timeline of clinical manifestations. Macrocephaly, spasticity, motor and cognitive regression, and psychiatric symptoms are the main clinical manifestations of the proband.

The proband also presented with severe motor developmental delay and low muscle tone. She could not roll over at 6 months, stand until she was 1 year and 6 months of age, or crawl and independently sit and walk until she was 2 years old. She often walked unsteadily and exhibited poor motor coordination. She had no early-onset seizures, epilepsy, or spasticity before 2 years of age, and her vision and hearing functions were normal. Her cognitive functions were delayed; she could say a few enunciated words but often spoke to herself unconsciously and could not understand the meaning of words, follow instructions, express her intentions, or otherwise verbally communicate. She had a motor developmental index of less than 50 on the Bayley Scales of Infant Development (BSID-II) at 19 months of age and a cognitive developmental index of 52, which, respectively, equaled those of 10- and 12-month-old infants. These results indicated that the patient had substantial deficits in her motor, cognitive, and language development levels outside the normal range. Detailed information is provided in Table 1; Figure 3.

**Table 1:** The Comparison of clinical findings in MLC.

Clinical findings of MLC1 reported previously	Clinical findings of the present patient (age of onset)	Clinical findings of the case with the same variant* (age of onset)
Macrocephaly/increasing HC	+(6 month)	+(3 month)
Brain MRI		
Subcortical cysts	−	+
Leukodystrophy	+	/
Cortical atrophy	−	/
Developmental delay	+	/
Motor function		
Independent walking	+(2 year)	+(2 year)
Ataxic gait	+	/
Motor deterioration	+(4 year)	+
Loss of ambulation	+(5 year)	+(5 year)
Speech		
First words	(1 year)	(1 year)
Speech deterioration	+(6 year)	/
Dysarthria	+(6 year)	/
Neurologic injuries		
Hypotonia/dystonia	+(6 month)	/
Pyramidal findings	+	/
Neuroregression	+	/
Seizures after head trauma(Focal epilepsy)	+(2.5 year)	+
Tremor	−	/
Choreoathetosis	−	/
Cognitive deterioration	+(6 year)	/
Emotional and behavioral problems	+(3 year)	+(4–5 year)
Hearing disorder	−	/
Visual impairment	−	/
Dysphagia	−	/
Absence of bladder control	−	/

“/”, not available; *Patrono et al. (10).

### 3.2. Clinical follow-up of the proband

We also followed the proband’s clinical course after she turned 2 years-old. After becoming 2 ½ years-old, the patient began to experience convulsions and epilepsy, accompanied by strabismus and loss of consciousness, lasting 1 to 2 min, which occurred after a collision on the top of her head. She has been taking valproate (depakin) since then, bringing her seizures under control with an average of 1–2 minor epileptic events per year. At 3 years of age, an MRI of the brain showed no obvious changes in brain lesions (Figure 2B); however, she began to experience deterioration in motor function; she could not walk unaided, although unsteadily, until she was 4 years old. After that, her motor functions progressively regressed, and she relied on a wheelchair after 5 years of age. Her language skills also did not improve, and her speech became slurred at 3 years old. From the age of 6 years, her cognitive function appeared worse than before; she began to have hypomnesia, forgetting songs she used to remember. She developed psychiatric problems at 3 years old and gradually began crying frequently and exhibiting more severe signs, including screaming, poor sleep, and difficulty regulating her emotions. She was prescribed olanzapine at 3 years of age, which reduced her crying to some extent. The parents also found ways to calm her, so they continued olanzapine treatment under the guidance of a psychiatrist. She had been undergoing regular rehabilitation training at a local rehabilitation center since the age of 5 years but to little avail. Electroencephalogram analysis at 8 years of age showed diffuse mixed slow-wave activity in the bilateral prefrontal, frontal, central, parietal, occipital, and temporal regions during awakening. Regrettably, as the child was emotional and unable to cooperate, valuable electroencephalogram data have not yet been collected, and she could not undergo a brain MRI again. Detailed information is provided in Figure 3; Table 1.

### 3.3. Analysis of the splice mutation in this family

A cerebral white matter disorder was suspected based on clinical features and MRI findings. To confirm the diagnosis of the proband, we screened for genetic mutations using a monogenic disorder panel associated with leukoencephalopathy.

**Figure 4:**
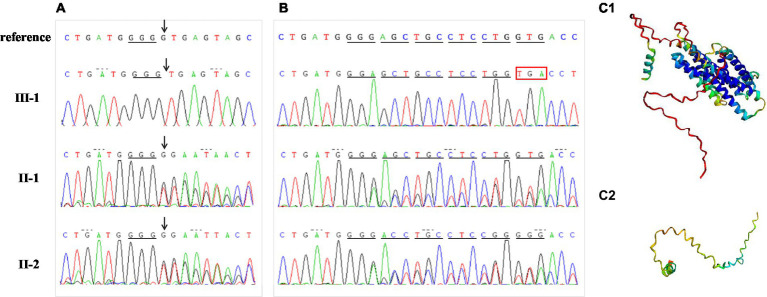
Mutation analysis. **(A)** Proband DNA sequencing (III-1) with an arrow pointing to the homozygous deletion (IVS2 + 1delG) of *MLC1*. Sequences of this mutation region in other family members (II-1, II-2) with an arrow pointing to heterozygous deletion. **(B)** Results of cDNA sequencing for the proband and her parents. Compared to normal protein translation, the homozygous single-base deletion (IVS2 + 1delG) changed the donor splice site, disrupted normal splicing, and caused a premature stop codon. **(C)** The protein structure was modeled by Alphafold2 in wild (C1) and mutant (C2) types, respectively.

A homozygous splice variant, NM_015166.4: [IVS2 + 1 delG], of *MLC1,* was identified in the proband (Figures 1A, 4A). These results were confirmed by Sanger sequencing, and the patient was verified to be homozygous for a mutation passed down from her parents and grandmothers (Figure 1A). None of the 100 Chinese normal controls harbored this mutation. This mutation was not found in the ClinVar database or reported in the literature, nor was it included in the healthy population (gnomAD, the 1,000 Genomes Project), local, or SNP databases. No other plausible candidate variants were consistent with the clinical phenotype of the patient.

“Deletion G” is in a classical splice recognition site, which can influence mRNA splicing, so the possibility of the disease being caused by a homozygous mutation cannot be ruled out, and no functional studies have confirmed the reliability of its pathogenicity. Therefore, we conducted further functional analyses by examining mRNA reverse transcription and predicting the protein structure. The cDNA of nuclear family members (parents and the proband) was obtained using RT-PCR. The cDNA amplicon sequence indicated that the mutation was responsible for the protein truncation of the *MLC1* gene after exon 2 (Figure 4B). The homozygous single-base deletion disrupts the normal donor splice site of intron 2, which inhibits splicing and causes an aberrant transcript that retains intron 2. A reading frame shift occurs at the codon from GGG to GGA, and the translation continues for five amino acids; finally, it is terminated early after reaching a premature stop codon (TGA; Figure 4B). The wild-type protein structures and of this mutation were predicted using Alphafold2, indicating that the mutation prematurely terminates the synthesis of the *MLC1* protein, preventing the formation of a normal three-dimensional protein structure as illustrated in Figure 4C. Consequently, this variant likely leads to a loss of function of *MLC1*.

### 3.4. Genetic counseling and prenatal diagnosis

**Figure 5:**
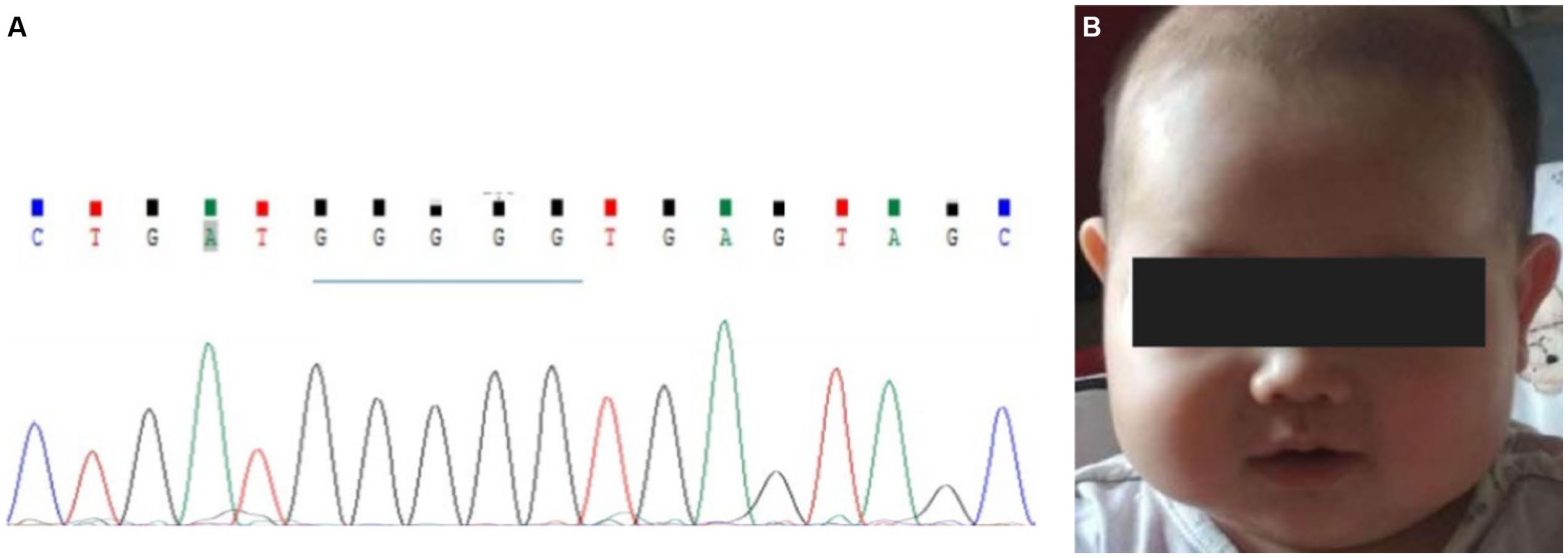
Prenatal diagnosis for the second child. **(A)** gDNA sequence of amniotic fluid cells. The arrow shows the position of the normal site, IVS2 + 1. **(B)** No abnormalities were found when the baby was approximately 10 months old.

Molecular genetic tests and functional analysis confirmed the pathogenicity of the *MLC1* variant IVS2 + 1 delG(PVS1 + PS3 + PM2) (11), and genetic counseling was performed to assess the risk of recurrence and provide guidance on fertility. We informed the couple that they had a 25% risk of having another child with abnormalities. The gravida received a molecular prenatal diagnosis by amniocentesis of DNA extracted in the 19th week of pregnancy, confirming that the fetus possessed the wild type of this site (Figure 5A). Therefore, we recommended the gravida continue the pregnancy. The following year, a healthy baby was born at full term (Figure 5B). The second child never showed similar symptoms and signs as his older sister, and all of his physical indicators were normal, including HC.

## 4. Discussion

An increasing number of patients have been diagnosed with MLC globally, although the clinical manifestations of MLC vary greatly. Leukoencephalopathy is distinguished by the inconsistency between mild neurological findings and severe white matter abnormalities (3, 12–14). The patient exhibited typical clinical manifestations, such as a substantially increased HC, severely delayed motor development, and diffuse symmetrical white matter lesions according to brain MRI scans, which are summarized in Table 1.

The hallmark of MLC is the presence of subcortical cysts in the anterior temporal, frontal, or parietal regions (2, 15). Brain biopsies from a patient with MLC showed vacuolization of myelin (16), and the vacuoles were lined with myelin, representing intramyelinic edema, and no loss of myelin (17). Duarri et al. (18) established an *in vitro* MLC model in which *MLC1* was knocked down in primary astrocytes; reduced expression of *MLC1* resulted in the appearance of intracellular vacuoles, proposing that this vacuolization is associated with the loss of *MLC1*’s role in maintaining integrity. Interestingly, previously reported patients with *MLC1* mutations showed more subcortical cysts outside the temporal region than those with *HEPACAM* mutations (2, 19). However, the brain MRI of this proband was not entirely similar to those previously reported, as subcortical cysts were not found at the ages of 2 and 3 years. A previous study reported two sisters with compound heterozygous mutations (c. 393C > T and c. 823C > A) in *MLC1*, and subcortical cysts were not found in all sections (axial, coronal, sagittal image) of the brain MRI of the older sister (20), similar to the MRI findings of this patient. Mahmoud et al. reported manifold brain MRI findings, including mild cerebellar vermis hypoplasia and mild ventriculomegaly. Furthermore, inter and intrafamilial variability has been observed (19, 21), revealing that some unknown modifying factors can influence the phenotype. Thus, more studies are necessary on the specific functions of *MLC1* to verify the link between *MLC1* and subcortical cysts.

Macrocephaly is the most consistent and prominent feature observed in patients with MLC. The proband had a large HC (53.5 cm, > + 3SD) from 6 to 15 months of age. As seen in the HC curve of this proband (Figure 1C), the rapid early growth of the head, crossing the +3SD at approximately 6 months, was only followed a few months later (at approximately 1 year of age) by slow growth, which parallels the median curve. The change trend of the HC curve in the early stage was roughly consistent with that previously reported (12). Motor impairment is also one of the most common manifestations of MLC. The proband showed progressive deterioration of gait by her 4th year, and she finally had to rely on a wheelchair after the age of 5 years, although Patrono et al. observed a stationary course in patients who could not walk and showed no signs of deterioration (10, 21).

Epilepsy is a common feature in patients with MLC. Research has shown that the regulatory disorder of astrocytes in the homeostasis of ions and water leads to overexcitation of neural networks and seizures (22). The onset of epilepsy does not appear to depend on the number of cysts or the causative gene (21). This proband began to have generalized tonic–clonic seizures after 2 years of age, accompanied by strabismus and loss of consciousness, which were provoked by minor head trauma, as reported in the literature (23). She responded well to antiepileptic drugs and her seizures were controlled and reduced to an average of one to two minor epileptic episodes per year. Furthermore, mild-to-moderate developmental delays have been reported in patients with MLC of various ethnic backgrounds (3, 10, 12, 19, 24, 25). This proband experienced an obvious stagnation period in the development of her language skills and cognitive function, after which they retrogressed. The proband is currently 10 years old, her height and weight are within the normal range, and she has no growth restriction. However, it is unknown whether this situation will change after she enters puberty.

The non-specific clinical manifestations and imaging features of the proband suggested the possibility of leukodystrophy, which led to a bottleneck in her clinical diagnosis. It is a challenge to provide pregnant women with effective genetic counseling and an accurate risk assessment. Advances in molecular diagnostic tools have allowed us to identify the detailed underlying etiology of leukodystrophy and to increase the diagnostic yield (26). Therefore, we performed exome sequencing for the target gene panel associated with leukoencephalopathy. The *MLC1* mutation IVS2 + 1delG identified in this study is a homozygous mutation found in both parents with no consanguinity and is not a uniparental diploid. Although this mutation was recorded in the HGMD database as c.177 + 1delG (10, 27), the description of this mutation was indistinct in these two studies, and the effect of changes in RNA splicing was not confirmed by transcriptional studies. Therefore, in this study, for an accurate prenatal diagnosis, we analyzed the family cDNA of the mutant gene by RT-PCR and confirmed that the mutation caused changes in mRNA splicing. Splicing mutations can cause the retention of introns, complete skipping of exons, or the introduction of a new splice site within an exon or intron (28). This variant disrupted the donor splice site of intron 2, inhibited splicing, resulted in the retention of intron 2, and produced an aberrant transcript (Figure 4B). This variation in *MLC1* may produce a truncated protein without the normal eight transmembrane domains (6, 7) or induce degradation of mRNAs by nonsense-mediated mRNA decay, which was also indicated by the protein structure predicted by Alphafold2. Having elucidated the function of this genetic variation, we performed a prenatal diagnosis of the fetus.

There is no cure for MLC, and treatment is primarily symptomatic. Bosch et al. injected an adeno-associated virus (AAV), encoding human *MLC1* under the control of a glial fibrillary acid protein promoter into the subarachnoid space of the cerebellum of *Mlc1* knockout mice (29). The expression of *MLC1* in the cerebellum significantly reduced myelin vacuolation at all ages in a dose-dependent manner. This study may provide patients with MLC with a potential therapeutic approach in the future.

In conclusion, we analyzed a variant of *MLC1* and confirmed that it is pathogenic, probably causing MLC. In addition, the clinical course of the patient and the findings of the brain MRI are detailed. Furthermore, we made a prenatal diagnosis for this family on the explicit basis of the proband. Our study provides additional information on the genotype and atypical phenotype of MLC1 and the prenatal diagnosis process, which is important for physicians providing genetic counseling and conducting prenatal diagnosis or preimplantation genetic tests for families. The information and clinical experience in this study could contribute to the field, and help other families with MLC make informed decisions about their reproductive options.

## Data availability statement

The datasets presented in this article are not readily available because of ethical and privacy restrictions. Requests to access the datasets should be directed to the corresponding author.

## Ethics statement

The studies involving human participants were reviewed and approved by the Medical Ethical Committees of the West China Second University Hospital, Sichuan University. Written informed consent to participate in this study was provided by the participants' legal guardian/next of kin. Written informed consent was obtained from the individual(s), and minor(s)' legal guardian/next of kin, for the publication of any potentially identifiable images or data included in this article.

## Author contributions

XCh: Writing – original draft. HQ: Writing – review & editing. QY: Writing – review & editing. XCa: Writing – review & editing. TH: Writing – review & editing. XZ: Writing – review & editing.

## Footnotes

^1^https://github.com/deepmind/alphafold

## References

[1] López-Hernández T Sirisi S Capdevila-Nortes X Montolio M Fernández-Dueñas V Scheper GC. Molecular mechanisms of MLC1 and GLIALCAM mutations in megalencephalic leukoencephalopathy with subcortical cysts. Hum Mol Genet. (2011) 20:3266–77. doi: 10.1093/hmg/ddr238, PMID: 21624973

[2] van der Knaap MS Boor I Estevez R. Megalencephalic leukoencephalopathy with subcortical cysts: chronic white matter oedema due to a defect in brain ion and water homoeostasis. Lancet Neurol. (2012) 11:973–85. doi: 10.1016/S1474-4422(12)70192-8, PMID: 23079554

[3] van der Knaap MS Barth PG Stroink H van Nieuwenhuizen O Arts WFM Hoogenraad F. Leukoencephalopathy with swelling and a discrepantly mild clinical course in eight children. Ann Neurol. (1995) 37:324–34. doi: 10.1002/ana.410370308, PMID: 7695231

[4] Durand CM Betancur C Boeckers TM Bockmann J Chaste P Fauchereau F. Mutations in the gene encoding the synaptic scaffolding protein SHANK3 are associated with autism spectrum disorders. Nat Genet. (2007) 39:25–7. doi: 10.1038/ng1933, PMID: 17173049 PMC2082049

[5] Nomura N Miyajima N Sazuka T Tanaka A Kawarabayasi Y Sato S. Prediction of the coding sequences of unidentified human genes. I. the coding sequences of 40 new genes (KIAA0001-KIAA0040) deduced by analysis of randomly sampled cDNA clones from human immature myeloid cell line KG-1. DNA Res. (1994) 1:27–35. doi: 10.1093/dnares/1.1.27, PMID: 7584026

[6] Lanciotti A Brignone MS Molinari P Visentin S de Nuccio C Macchia G. Megalencephalic leukoencephalopathy with subcortical cysts protein 1 functionally cooperates with the TRPV4 cation channel to activate the response of astrocytes to osmotic stress: dysregulation by pathological mutations. Hum Mol Genet. (2012) 21:2166–80. doi: 10.1093/hmg/dds032, PMID: 22328087

[7] Leegwater PA Yuan BQ van der Steen J Mulders J Könst AAM Boor PKI. Mutations of MLC1 (KIAA0027), encoding a putative membrane protein, cause megalencephalic leukoencephalopathy with subcortical cysts. Am J Hum Genet. (2001) 68:831–8. doi: 10.1086/319519, PMID: 11254442 PMC1275636

[8] Liu J Gao J Jiang Y. Invasive prenatal diagnostic techniques: From theory to practice. 1st ed. Beijing: People's Military Medical Press (2012). 9 p.

[9] National Health Commission of the People’s Republic of China. Growth standard for children under 7 years of age. (2022).

[10] Patrono C di Giacinto G Eymard-Pierre E Santorelli FM Rodriguez D de Stefano N. Genetic heterogeneity of megalencephalic leukoencephalopathy and subcortical cysts. Neurology. (2003) 61:534–7. doi: 10.1212/01.WNL.0000076184.21183.CA, PMID: 12939431

[11] Richards S Aziz N Bale S Bick D das S Gastier-Foster J. Standards and guidelines for the interpretation of sequence variants: a joint consensus recommendation of the American College of Medical Genetics and Genomics and the Association for Molecular Pathology. Genet Med. (2015) 17:405–24. doi: 10.1038/gim.2015.30, PMID: 25741868 PMC4544753

[12] Cao B Yan H Guo M Xie H Wu Y Gu Q. Ten novel mutations in Chinese patients with megalencephalic leukoencephalopathy with subcortical cysts and a long-term follow-up research. PLoS One. (2016) 11:e0157258. doi: 10.1371/journal.pone.0157258, PMID: 27322623 PMC4913951

[13] van der Knaap MS Lai V Köhler W Salih MA Fonseca MJ Benke TA. Megalencephalic leukoencephalopathy with cysts without MLC1 defect. Ann Neurol. (2010) 67:NA–7. doi: 10.1002/ana.21980, PMID: 20517947

[14] van der Knaap MS Valk J Barth PG Smit LME van Engelen BGM Donati PT. Leukoencephalopathy with swelling in children and adolescents: MRI patterns and differential diagnosis. Neuroradiology. (1995) 37:679–86. doi: 10.1007/BF00593394, PMID: 8748906

[15] van der Knaap MS Bugiani M. Leukodystrophies: a proposed classification system based on pathological changes and pathogenetic mechanisms. Acta Neuropathol. (2017) 134:351–82. doi: 10.1007/s00401-017-1739-1, PMID: 28638987 PMC5563342

[16] van der Knaap MS Barth PG Vrensen GF Valk J. Histopathology of an infantile-onset spongiform leukoencephalopathy with a discrepantly mild clinical course. Acta Neuropathol. (1996) 92:206–12. doi: 10.1007/s004010050510, PMID: 8841668

[17] van der Knaap MS Bugiani M. Leukodystrophies - much more than just diseases of myelin. Nat Rev Neurol. (2018) 14:747–8. doi: 10.1038/s41582-018-0093-9, PMID: 30341432

[18] Duarri A Lopez de Heredia M Capdevila-Nortes X Ridder MC Montolio M López-Hernández T. Knockdown of MLC1 in primary astrocytes causes cell vacuolation: a MLC disease cell model. Neurobiol Dis. (2011) 43:228–38. doi: 10.1016/j.nbd.2011.03.015, PMID: 21440627 PMC3885813

[19] Mahmoud IG Mahmoud M Refaat M Girgis M Waked N el Badawy A. Clinical, neuroimaging, and genetic characteristics of megalencephalic leukoencephalopathy with subcortical cysts in Egyptian patients. Pediatr Neurol. (2014) 50:140–8. doi: 10.1016/j.pediatrneurol.2013.10.008, PMID: 24315536

[20] Masuda T Ueda M Ueyama H Shimada S Ishizaki M Imamura S. Megalencephalic leukoencephalopathy with subcortical cysts caused by compound heterozygous mutations in MLC1, in patients with and without subcortical cysts in the brain. J Neurol Sci. (2015) 351:211–3. doi: 10.1016/j.jns.2015.03.010, PMID: 25796299

[21] Abdel-Salam GM Abdel-Hamid MS Ismail SI Hosny H Omar T Effat L. Megalencephalic leukoencephalopathy with cysts in twelve Egyptian patients: novel mutations in MLC1 and HEPACAM and a founder effect. Metab Brain Dis. (2016) 31:1171–9. doi: 10.1007/s11011-016-9861-7, PMID: 27389245

[22] Dubey M Brouwers E Hamilton EMC Stiedl O Bugiani M Koch H. Seizures and disturbed brain potassium dynamics in the leukodystrophy megalencephalic leukoencephalopathy with subcortical cysts. Ann Neurol. (2018) 83:636–49. doi: 10.1002/ana.25190, PMID: 29466841 PMC5900999

[23] Pascual-Castroviejo I van der Knaap MS Pronk JC García-Segura JM Gutiérrez-Molina M Pascual-Pascual SI. Vacuolating megalencephalic leukoencephalopathy: 24 year follow-up of two siblings. Neurologia. (2005) 20:33–40. PMID: 15704020

[24] Gorospe JR Singhal BS Kainu T Wu F Stephan D Trent J. Indian Agarwal megalencephalic leukodystrophy with cysts is caused by a common MLC1 mutation. Neurology. (2004) 62:878–82. doi: 10.1212/01.WNL.0000115106.88813.5B, PMID: 15037685

[25] Tinsa F Farid O Douira W Rodriguez D Burglen L Boussetta K. Megalencephalic leukoencephalopathy with subcortical cysts in a Tunisian boy. J Child Neurol. (2009) 24:87–9. doi: 10.1177/0883073808324021, PMID: 19168821

[26] Ashrafi MR Amanat M Garshasbi M Kameli R Nilipour Y Heidari M. An update on clinical, pathological, diagnostic, and therapeutic perspectives of childhood leukodystrophies. Expert Rev Neurother. (2020) 20:65–84. doi: 10.1080/14737175.2020.1699060, PMID: 31829048

[27] Chen X Qu H Yu T Luo R. Identification of a novel MLC1 mutation in a Chinese patient affected with megalencephalic leukoencephalopathy with subcortical cysts. Zhonghua Yi Xue Yi Chuan Xue Za Zhi. (2016) 33:316–9. doi: 10.3760/cma.j.issn.1003-9406.2016.03.008, PMID: 27264811

[28] Baralle D Baralle M. Splicing in action: assessing disease causing sequence changes. J Med Genet. (2005) 42:737–48. doi: 10.1136/jmg.2004.029538, PMID: 16199547 PMC1735933

[29] Bosch A Estevez R. Megalencephalic leukoencephalopathy: insights into pathophysiology and perspectives for therapy. Front Cell Neurosci. (2020) 14:627887. doi: 10.3389/fncel.2020.627887 33551753 PMC7862579

